# Predicting survival of advanced laryngeal squamous cell carcinoma: comparison of machine learning models and Cox regression models

**DOI:** 10.1038/s41598-023-45831-8

**Published:** 2023-10-28

**Authors:** Yi-Fan Zhang, Yu-Jie Shen, Qiang Huang, Chun-Ping Wu, Liang Zhou, Heng-Lei Ren

**Affiliations:** Department of Otorhinolaryngology, Eye & ENT Hospital, Fudan University, Shanghai, 200031 China

**Keywords:** Cancer models, Head and neck cancer

## Abstract

Laryngeal squamous cell carcinoma (LSCC) is a common tumor type. High recurrence rates remain an important factor affecting the survival and quality of life of advanced LSCC patients. We aimed to build a new nomogram and a random survival forest model using machine learning to predict the risk of LSCC progress. The study included 671 patients with AJCC stages III–IV LSCC. To develop a prognostic model, Cox regression analyses were used to assess the relationship between clinic-pathologic factors and disease-free survival (DFS). RSF analysis was also used to predict the DFS of LSCC patients. The ROC curve revealed that the Cox model exhibited good sensitivity and specificity in predicting DFS in the training and validation cohorts (1 year, validation AUC = 0.679, training AUC = 0.693; 3 years, validation AUC = 0.716, training AUC = 0.655; 5 years, validation AUC = 0.717, training AUC = 0.659). Random survival forest analysis showed that N stage, clinical stage, and postoperative chemoradiotherapy were prognostically significant variables associated with survival. The random forest model exhibited better prediction ability than the Cox regression model in the training cohort; however, the two models showed similar prediction ability in the validation cohort.

## Introduction

Head and neck squamous cell carcinoma (HNSCC) is the seventh most common cancer in the world. Asia has the highest incidence rate of head and neck cancer. The number of deaths due to head and neck cancer accounts for more than 5% of all cancer deaths^1^. Among these, laryngeal squamous cell carcinoma (LSCC) is one of the most common tumor types. In 2020, the number of new cases of laryngeal cancer worldwide exceeded 180,000^2^. Squamous cell carcinoma accounts for more than 90% of laryngeal carcinoma cases. At present, surgical treatment is the main treatment for LSCC. The main surgical options include laser surgery, partial laryngectomy, and total laryngectomy. It is difficult to retain the laryngeal function of advanced LSCC patients, and surgery will seriously affect or even destroy the patient’s voice, swallowing, and other functions. For patients with advanced laryngeal cancer with or without metastasis, radiotherapy/chemotherapy is an important adjuvant treatment^3^. Although the prognosis of laryngeal cancer patients is generally good, for patients with advanced LSCC, a high recurrence rate is still one of the important factors affecting survival and quality of life.

There are many survival prediction models for LSCC patients. A retrospective study included 84 LSCC cases revealed that recurrence and lymph invasion were the only factors that had an independent effect on OS and recurrence in DSS. Furthermore, subsite location was the only factor in multivariate analysis that impacted DFS and LRC^4^. Another study showed that survival outcomes of patients with well to moderately differentiated LSCCs were significantly better than those of patients with poorly differentiated tumors in DFS^5^. However, the prediction of the progression time for advanced LSCC patients is still relatively lacking. Random survival forest (RSF) models, one of the machine learning models, are increasingly being used in the building of predictive survival models^6^. Based on this background, we aimed to develop a novel nomogram and RSF models to predict the risk of progress in laryngeal carcinoma. Moreover, we will also compare the advantages and disadvantages of the two models.

## Methods

### Data source and study population

The study included 671 patients with American Joint Committee on Cancer (AJCC) stage III–IV LSCC treated at the Eye & ENT Hospital of Fudan University between October 2008 and June 2012. The inclusion criteria were as follows: (1) an operation was performed, and (2) the patient medical records were available. All patients were routinely followed up via postal letters and/or telephone interviews with patients and their relatives.

### Cox regression model establishment

To develop a prognostic model, univariate Cox regression and multivariate Cox regression analyses were used to assess the relationship between clinic-pathologic factors and disease-free survival (DFS). All clinic-pathologic factors were included in the univariate Cox regression. Variables with a P < 0.2 were identified for multivariate Cox regression analyses (70% training data and 30% out-of-sample data). Cox regressions were carried out using the survival package. The hazard ratio (HR) was used to interpret the risk of recurrence/metastasis in parametric results, and the effectiveness of models was evaluated using Harrell's concordance index (C-index). A P < 0.05 was considered statistically significant. The receiver operating characteristic (ROC) curve was implemented using the R software package survival ROC. A nomogram was constructed using the R software package *regplot*.

### Random survival forest model

The disease-free survival of patients with Laryngeal Squamous Cell Carcinoma (LSCC) was predicted using the random Forest SRC package in R software, through the implementation of RSF analysis. The dataset was separated into 70% training data and 30% out-of-sample data. The cohort was split into training and validation cohort using “sample” package in R software, and the seed was set as 123. *ntree* was set at 500. Harrell’s concordance index was used to calculate the accuracy of the model. VIMP is used to describe the importance of a variable (a variable with a VIMP value less than 0 indicates that the variable reduces the accuracy of the prediction, while a VIMP value greater than 0 indicates that the variable improves the accuracy of the prediction).

### Ethics statement

All participants provided written informed consent. The protocols were authorized by the experimental protocol was established, according to the ethical guidelines of the Helsinki Declaration and was approved by the Clinical Research Ethics Committee of the Eye & ENT Hospital of Fudan University (No. KJ2008-01). Written informed consent was obtained from a legally authorized representatives for anonymized patient information to be published in this article.

## Results

### Baseline characteristic analysis of patients

**Figure 1:**
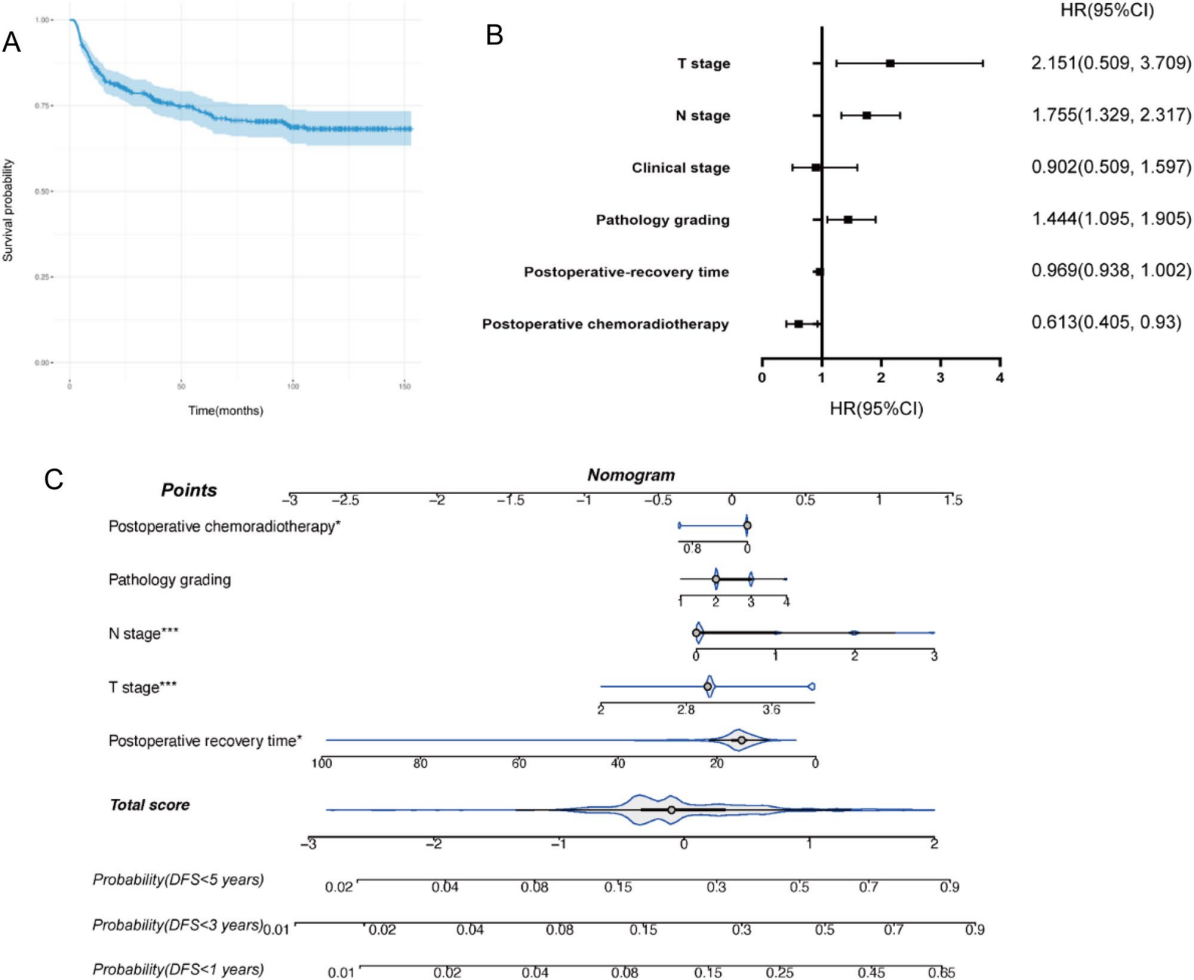
(**A**) Kaplan–Meier curves pertaining to disease-free survival (DFS). (**B**) Forest plot of DFS. (**C**) Nomogram of DFS.

A total of 671 patients with advanced LSCC (AJCC stages III–IV) were included in this study. For statistical analysis, all patients were divided into two groups according to whether disease progression (recurrence/metastasis) occurred during follow-up. The analysis indicated that T stage, clinical stage, N stage, volume of tumor, and resection margins were significantly associated with the progression of LSCC (Table 1). The overall progression-free rate of the patients was 73.7% (Fig. 1A).

**Table 1:** Clinical factors of 671 advanced LSCC patients.

Factors		N	Non progress	Progress	P-value
Gender					0.121
	Male	655	480	175	
	Female	16	15	1	
Age					0.583
	≤ 60	336	251	85	
	> 60	335	244	91	
Postoperative time					0.139
	< 12	50	38	12	
	≥ 12	621	457	164	
Marriage					0.292
	Married	650	478	172	
	Unmarried	7	5	2	
	Widowed	5	3	2	
	Divorced	9	9	0	
Smoke					0.613
	No	158	119	39	
	Yes	513	376	137	
Alcohol					0.373
	No	351	264	87	
	Yes	320	231	89	
Hypertension/diabetes					0.34
	No	517	390	127	
	Hypertension	115	79	36	
	Diabetes	25	17	8	
	Hypertension + diabetes	14	9	5	
Preoperative chemoradiotherapy					0.712
	No	657	484	173	
	Radiotherapy	13	10	3	
	Chemoradiotherapy	1	1	0	
Surgery					0.789
	Total laryngectomy	537	390	147	
	Vertical partial laryngectomy	63	49	14	
	Horizontal partial laryngectomy	12	10	2	
	CHEP	48	37	11	
	CHP	10	8	2	
	CO_2_ laser laryngeal tumor resection	1	1	0	
Neck lymph node dissection					0.156
	No	416	320	96	
	Unilateral selective neck dissection	56	38	18	
	Unilateral radical neck dissection	151	104	47	
	Bilateral selective neck dissection	39	28	11	
	Unilateral radical neck dissection+ unilateral selective neck dissection	9	5	4	
Primary site					0.366
	Supraglottic	245	173	72	
	Glottic	417	315	102	
	Subglottic	9	7	2	
T stage					**0.002**
	2	2	2	0	
	3	515	396	119	
	4	154	97	57	
N stage					**0.001**
	0	498	387	111	
	1	61	44	17	
	2	96	56	40	
	3	16	8	8	
Clinical stage					**0.001**
	III	434	345	89	
	IVa	222	143	79	
	IVb	15	7	8	
Volume					**0.007**
	<2.7	344	269	75	
	>2.7	327	226	101	
Resection margins					**0.001**
	>0.5	488	367	121	
	<0.5	156	102	54	
	Positive	27	26	1	
Pathology grading					0.178
	I	1	0	1	
	II	390	293	97	
	I–II	257	188	69	
	III	23	14	9	
Postoperative chemoradiotherapy					0.219
	No	519	377	142	
	Yes	152	118	34	

Significant values are in bold.

### Cox regression modeling process and nomogram construction

A training cohort was used to assess the prognostic importance of each component in predicting DFS. Factors including T stage, N stage, clinical stage, volume of tumor, and neck dissection all had statistically significant predictive value in univariate Cox analyses (Table 2). For further multivariable Cox analysis, variables with P < 0.2 were selected. Thus, T stage, N stage, pathology grading, postoperative chemoradiotherapy, and postoperative recovery time were included in the prognostic model (Table 3). All significant variables were assessed using HR (Fig. 1B). The prognostic model is visually presented with a dynamic nomogram (Fig. 1C).

**Table 2:** uniCox factors with P-value<0.2.

Factors	SE	P	Hazard ratio	Lower (95% CI)	Upper (95% CI)
Clinical stage	0.131	0.000	2.117	1.371	2.598
N stage	0.078	0.000	1.527	1.195	2.032
T stage	0.160	0.000	1.924	1.235	3.672
Volume	0.153	0.003	1.581	1.061	1.948
Neck dissection			1.199		
No	0.066	0.006	1.00	0.999	1.401
Unilateral selective neck dissection	0.416	0.024	0.415	0.182	0.918
Unilateral radical neck dissection	0.459	0.104	0.475	0.193	1.166
Bilateral selective neck dissection	0.425	0.218	0.592	0.258	1.362
Unilateral radical neck dissection+ unilateral selective neck dissection	0.459	0.648	0.811	0.330	1.993
Hypertension/diabetes	0.103	0.093	1.189	0.969	1.423
Gender	1.003	0.117	4.819	0.642	8.906
Surgery	0.087	0.137	0.878	0.728	1.021
Primary site	0.148	0.139	0.804	0.604	1.100
Supraglottic			1.00		
Glottic	0.409	0.494	1.124	0.215	1.952
Subglottic	0.406	0.617	1.015	0.277	2.035
Pathology grading	0.122	0.181	1.171	0.923	1.397
Postoperative chemoradiotherapy	0.191	0.196	0.807	0.478	1.236

**Table 3:** multiCox factors with P-value<0.05.

Factors	SE	P	Hazard ratio	Lower (95% CI)	Upper (95% CI)
N stage	0.142	0.001	1.755	1.329	2.317
T stage	0.278	0.006	2.151	1.248	3.709
Pathology grading	0.141	0.009	1.444	1.095	1.905
Postoperative recovery time	0.017	0.044	0.969	0.938	1.002
Postoperative chemoradiotherapy	0.212	0.021	0.613	0.405	0.93

### Cox regression model validation

**Figure 2:**
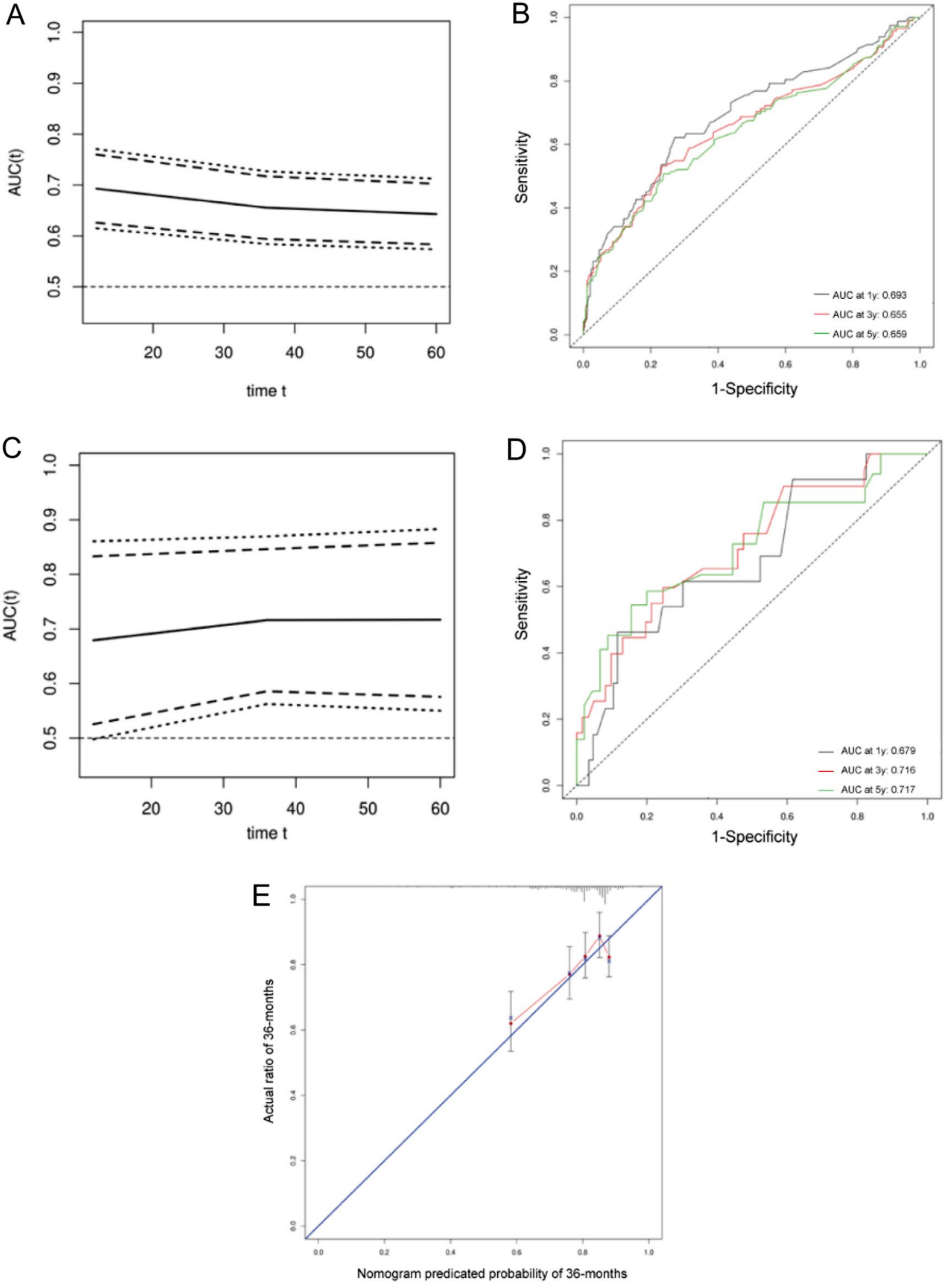
(**A**,**B**) The area under the curve (AUC) for Cox regression model predicting the DFS of training cohort. (**C**,**D**) the area under the curve (AUC) for Cox regression model predicting the DFS of validation cohort. (**E**) Calibration curve for 3-year DFS.

Using the validation cohort, the nomogram’s validation and evaluation were carried out. The prognostic model’s C-index was 0.656 (95% CI 0.598, 0.694), which was higher than any single factor or the TNM staging method (C-index: 0.603). ROC analysis, which explored the efficacy of the model, revealed that our model exhibited good sensitivity and specificity in predicting DFS in the training and validation cohorts (1 year, validation AUC = 0.679, training AUC = 0.693; 3 years, validation AUC = 0.716, training AUC = 0.655; 5 years, validation AUC = 0.717, training AUC = 0.659) (Fig. 2).

### Random survival modeling process and validation

**Figure 3:**
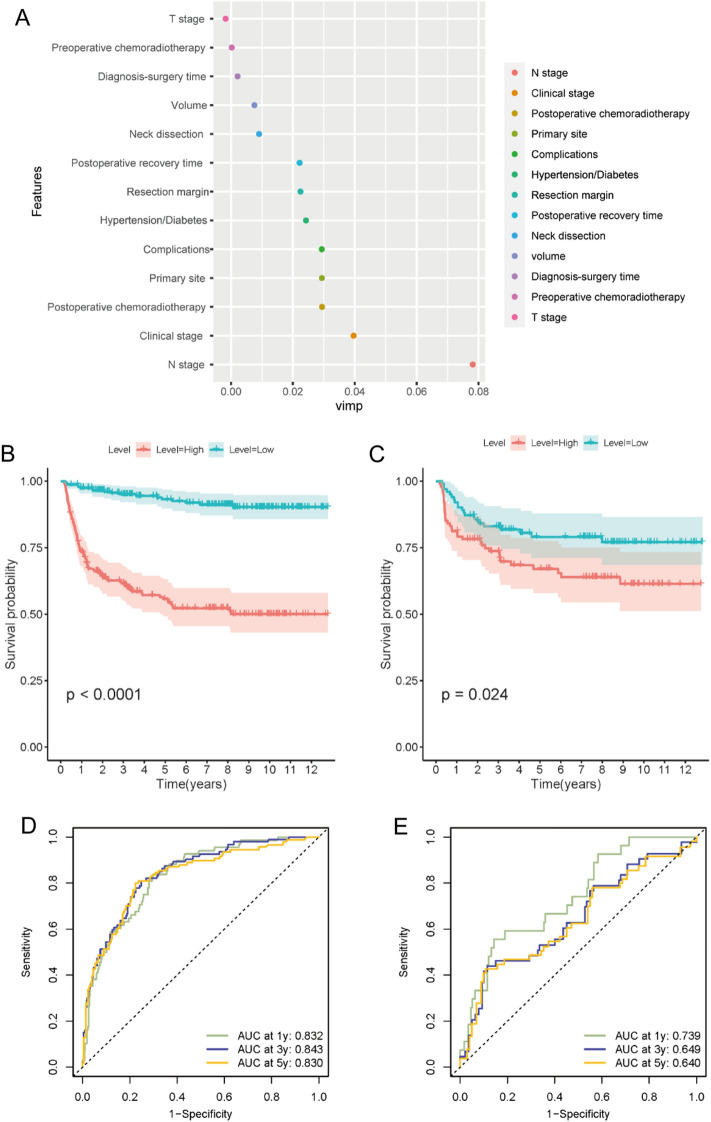
(**A**) VIMP of clinical factors in random survival forest model. (**B**) Kaplan-Meier survival curves of the high and low risk groups in training sets (P < 0.0001). (**C**) Kaplan-Meier survival curves of the high and low risk groups in validation sets (P = 0.0.024). (**D**) The ROC curve of predicting DFS in the training cohort. (**E**) The ROC curve of predicting DFS in the validation cohort.

The ensemble type classification method known as random forest (RF) typically outperforms more established decision tree classification techniques^7^. The survivorship prediction is based on the majority voting mechanism used by each tree. We employed 500 trees to forecast two target classes of advanced LSCC patients’ progress or nonprogress in the training cohort. VIMP analysis showed that N stage, clinical stage, and postoperative chemoradiotherapy were prognostically significant variables associated with survival (Fig. 3A). In both the training and validation sets, the Kaplan-Meier survival curves of the high and low risk groups were significantly different (P < 0.05) (Fig. 3B,C). The ROC curve revealed that the model exhibited good sensitivity and specificity in predicting DFS in the training cohort. However, the model exhibited suboptimal performance in the validation cohort (1 year, validation *AUC* = 0.739, training AUC = 0.832; 3 years, validation *AUC* = 0.649, training AUC = 0.843; 5 year, validation *AUC* = 0.640, training AUC = 0.830) (Fig. 3D,E)

## Discussion

Because of the variety of clinical characteristics and therapy options, the survival outcomes of LSCC vary among patients. Based on data from 671 patients with advanced LSCC, we developed the first machine learning model to predict DFS in advanced LSCC patients. The Cox regression model and random survival forest both showed good predictive ability.

Although HNSCC have great similarities in treatment, their clinical outcomes differ greatly. The lack of identifiable early signs in LSCC makes early detection of HSCC more difficult. In most countries, laryngoscopy is not a routine medical exam^8^. Thus, many LSCC patients have been confirmed to have advanced-stage disease at the initial diagnosis. Although patients with LSCC have a good prognosis after surgery and adjuvant treatment, postsurgical tumor recurrence and metastases remain major concerns for patients with advanced LSCC^9^.

Recently, a number of nomograms for predicting risk have been reported. In 2017, the Multidisciplinary Larynx Cancer Working Group developed a dynamic risk model and clinical nomogram for patients with locally advanced laryngeal cancer, utilizing conditional survival analysis and data from the University of Texas MD Anderson Cancer Center database^10^. In line with our findings, they found that nodal burden was an important factor for 3- or 6-year overall survival (OS) in the multivariate analysis. Shi et al. created another risk prediction model using data from 2752 LSCC patients who underwent neck dissection and were recorded in the Surveillance, Epidemiology, and End Results (SEER) database between 1988 and 2008^11^. The nomogram was constructed according to eight independent prognostic clinical variables. This study showed that the nomograms were superior to no-LNR (lymph node ratio) system and TNM classification. However, the accuracy of the prediction was probably reduced by the fact that only 20 patients were in the undifferentiated subset. Since then, Lin et al. established a prognostic model for advanced LSCC patients treated with primary total laryngectomy^12^, using an analysis data set collected from the SEER database. They identified six independent prognostic clinical variables. The C-index of the model was 0.651, which was similar to our model. Cui J al. constructed a survival prediction nomogram based on the data set including 369 patients with LSCC^13^. Six independent parameters predicting prognosis were age, pack-years, N stage, lymph node ratio (LNR), anaemia and albumin. The C-index of the nomogram was 0.73 (0.68–0.78), and the area under the curve (AUC) of the nomogram in predicting overall survival (OS) was 0.766.

In the current study, the first RSF prognostic model predicting DFS for advanced LSCC patients was built. We constructed a nomogram and an RSF model for predicting LSCC. Although the RSF model exhibited better prediction ability than the Cox regression model in the training cohort, both models showed similar prediction ability in the validation cohort. As a widely used machine learning model, the RSF model can judge the importance of factors without dimension reduction or feature selection. It can also judge the interactions between different features. However, RSF has been proven to be overfitting in some noisy classification or regression problems^14^. In our study, RSF exhibited significantly good sensitivity and specificity in the training cohort, although not in the validation cohort. We suspect that there are several possible reasons. First, our research data volume is not large, and the random forest model performs better in solving big data problems^15^. Another possible reason is some overfitting of the RSF model.

In the multivariable Cox regression model, we identified five independent predictors: T stage, N stage, postoperative chemoradiotherapy, pathology grading, and postoperative recovery time. The RSF model considered N stage, clinical stage, and postoperative chemoradiotherapy to be the three most important variables. Interestingly, T stage was a significant prognostic factor in the Cox model, although it was not identified as a significant prognostic variable in the RSF model. One possible reason was that the sample size was not large enough (Supplementary Table).

The nomogram and RSF models also revealed that adjuvant treatment is essential for prolonging the survival time of advanced LSCC patients. For patients with advanced LSCC, total laryngectomy is the standard treatment. According to NCCN guidelines, a remarkable amount of evidence showed significantly improved OS, disease-free survival, and locoregional control when a systemic therapy and radiation regimen (concomitant or, less commonly, sequential) was compared with RT alone for locoregionally advanced disease^16^. In a previous study, our research group reported that in patients with stage IV LSCC, those receiving adjuvant chemoradiotherapy exhibited a markedly improved survival benefit compared with patients receiving surgical treatment only^17^. Notably, in the present study, postoperative recovery time was identified as a significant variable in both the nomogram and RSF. Postoperative recovery time was strongly associated with clinical stage and surgery. Patients with a higher clinical stage and larger surgical range may need a longer time to recover.

Our study has several limitations. First, this was a retrospective study including LSCC patients undergoing laryngectomy only. As the treatment decision was made before inclusion in the study, there was a potential selection bias. Furthermore, our nomogram has not been applied to the prediction of survival in LSCC patients with other radical treatment models, such as radiotherapy and chemotherapy. Second, although the novel nomogram was generated based on a relatively large sample size and a split validation of the model was performed, no external validation using data from other centres was performed. Finally, only the clinicopathological prognostic factors were used to predict the survival rate. Hence, the decisions offered by the RSF model would be more comprehensive if both the clinicopathological and genomic data of LSCC patients were analyzed together.

### Supplementary Information

Supplementary Information.

## Data Availability

The datasets generated and/or analysed during the current study are not publicly available due to data containing private patient information but are available from the corresponding author on reasonable request.

## Abbreviations

HNSCC: Head and neck squamous cell carcinoma
LSCC: Laryngeal squamous cell carcinoma
AJCC: American Joint Committee on Cancer
RSF: Random survival forest
RF: Random forest
ROC: Receiver operating characteristic
AUC: Area under curve
OS: Overall survival
SEER: Surveillance, epidemiology, and end results
CHEP: Crico—hyoido—epiglotto—pexy
CHP: Crico—hyoido—pexy
LNR: Lymph node ratio
