# Supplementary material for: Predicting survival of advanced laryngeal squamous cell carcinoma: comparison of machine learning models and Cox regression models
Source: Sci Rep. 2023 Oct 28;13:18498. doi: 10.1038/s41598-023-45831-8 (PMC10613248; doi:10.1038/s41598-023-45831-8)
Supplement: Supplementary file 1 — Supplementary Information. [file 41598_2023_45831_MOESM1_ESM.docx]

Supplementary Table. clinical factors of 671 LSCC patients in training and validation cohort

| Factors |  | n | Training | Validation | P-value |
| --- | --- | --- | --- | --- | --- |
| Events |  |  |  |  | 0.703 |
|  | nonprogress | 495 | 348 | 147 |  |
|  | progress | 176 | 121 | 55 |  |
| Gender |  |  |  |  | 0.415 |
|  | Male | 655 | 456 | 199 |  |
|  | Female | 16 | 13 | 3 |  |
| Age |  |  |  |  | 0.239 |
|  | ≤60 | 336 | 242 | 94 |  |
|  | >60 | 335 | 227 | 108 |  |
| Postoperative time |  |  |  |  | 0.423 |
|  | <12 | 50 | 38 | 12 |  |
|  | ≥12 | 621 | 431 | 190 |  |
| Marriage |  |  |  |  | 0.292 |
|  | Married | 650 | 452 | 198 |  |
|  | Unmarried | 7 | 5 | 2 |  |
|  | Widowed | 5 | 4 | 1 |  |
|  | Divorced | 9 | 8 | 1 |  |
| Smoke |  |  |  |  | 0.321 |
|  | No | 158 | 105 | 53 |  |
|  | Yes | 513 | 364 | 149 |  |
| Alcohol |  |  |  |  | 0.556 |
|  | No | 351 | 249 | 102 |  |
|  | Yes | 320 | 220 | 100 |  |
| Hypertension/Diabetes |  |  |  |  | 0.161 |
|  | No | 517 | 362 | 155 |  |
|  | Hypertension | 115 | 75 | 40 |  |
|  | Diabetes | 25 | 19 | 6 |  |
|  | Hypertension + Diabetes | 14 | 13 | 1 |  |
| Preoperative chemoradiotherapy |  |  |  |  | 0.648 |
|  | No | 657 | 460 | 197 |  |
|  | Radiotherapy | 13 | 8 | 5 |  |
|  | Chemoradiotherapy | 1 | 1 | 0 |  |
| Surgery |  |  |  |  | 0.545 |
|  | Total laryngectomy | 537 | 375 | 162 |  |
|  | Vertical partial laryngectomy | 63 | 45 | 18 |  |
|  | Horizontal partial laryngectomy | 12 | 9 | 3 |  |
|  | CHEP | 48 | 35 | 13 |  |
|  | CHP | 10 | 5 | 5 |  |
|  | CO_2_ laser laryngeal tumor resection | 1 | 0 | 1 |  |
| Neck lymph node dissection |  |  |  |  | 0.524 |
|  | No | 416 | 297 | 119 |  |
|  | Unilateral selective neck dissection | 56 | 36 | 20 |  |
|  | Unilateral radical neck dissection | 151 | 100 | 51 |  |
|  | Bilateral selective neck dissection | 39 | 30 | 9 |  |
|  | Unilateral radical neck dissection+ unilateral selective neck dissection | 9 | 6 | 3 |  |
| Primary site |  |  |  |  | 0.354 |
|  | Supraglottic | 245 | 167 | 78 |  |
|  | Glottic | 417 | 295 | 122 |  |
|  | Subglottic | 9 | 7 | 2 |  |
| T stage |  |  |  |  |  |
|  | 2 | 2 | 2 | 0 |  |
|  | 3 | 515 | 365 | 150 |  |
|  | 4 | 154 | 102 | 52 |  |
| N stage |  |  |  |  | 0.143 |
|  | 0 | 498 | 349 | 149 |  |
|  | 1 | 61 | 39 | 22 |  |
|  | 2 | 96 | 66 | 30 |  |
|  | 3 | 16 | 15 | 1 |  |
| Clinical stage |  |  |  |  | 0.149 |
|  | III | 434 | 305 | 129 |  |
|  | IVa | 222 | 149 | 73 |  |
|  | IVb | 15 | 14 | 1 |  |
| Volume |  |  |  |  | 0.275 |
|  | <2.7 | 344 | 247 | 222 |  |
|  | >2.7 | 327 | 97 | 105 |  |
| Resection margins |  |  |  |  | 0.592 |
|  | >0.5 | 488 | 337 | 151 |  |
|  | <0.5 | 156 | 114 | 42 |  |
|  | Positive | 27 | 18 | 9 |  |
| Pathology grading |  |  |  |  | 0.918 |
|  | I | 1 | 1 | 0 |  |
|  | II | 390 | 274 | 116 |  |
|  | I-II | 257 | 178 | 79 |  |
|  | III | 23 | 16 | 7 |  |
| Postoperative chemoradiotherapy |  |  |  |  | 0.764 |
|  | No | 519 | 361 | 158 |  |
|  | Yes | 152 | 108 | 44 |  |
